# Orbito-facial plexiform neurofibromatosis: a rare clinical image

**DOI:** 10.11604/pamj.2023.44.25.37895

**Published:** 2023-01-12

**Authors:** Avi Sharma, Sachin Daigavane

**Affiliations:** 1 Department of Ophthalmology, Datta Meghe Institute of Medical Sciences, Sawangi (Meghe), Wardha, Maharashtra 442004, India

**Keywords:** Ophthalmology, neurosurgery, dermatology

## Image in medicine

An 18-year-old male reported chief complaints of inability to close his right eye. He is a known case of oro-facial plexiform neurofibromatosis for the past 12 years. The patient had undergone a debulking surgery in the right eyelid 2 days back. The patient's left eye had been enucleated 10 years ago. On ocular examination, the right upper eyelid showed upper eyelid mass with 6 sutures post debulking on the upper eyelid with scleral show. The left eye had been enucleated and tarsorrhaphy sutures were present. The patient was managed with topical lubricating eye drops and gel as well as lid patching.

**Figure 1:**
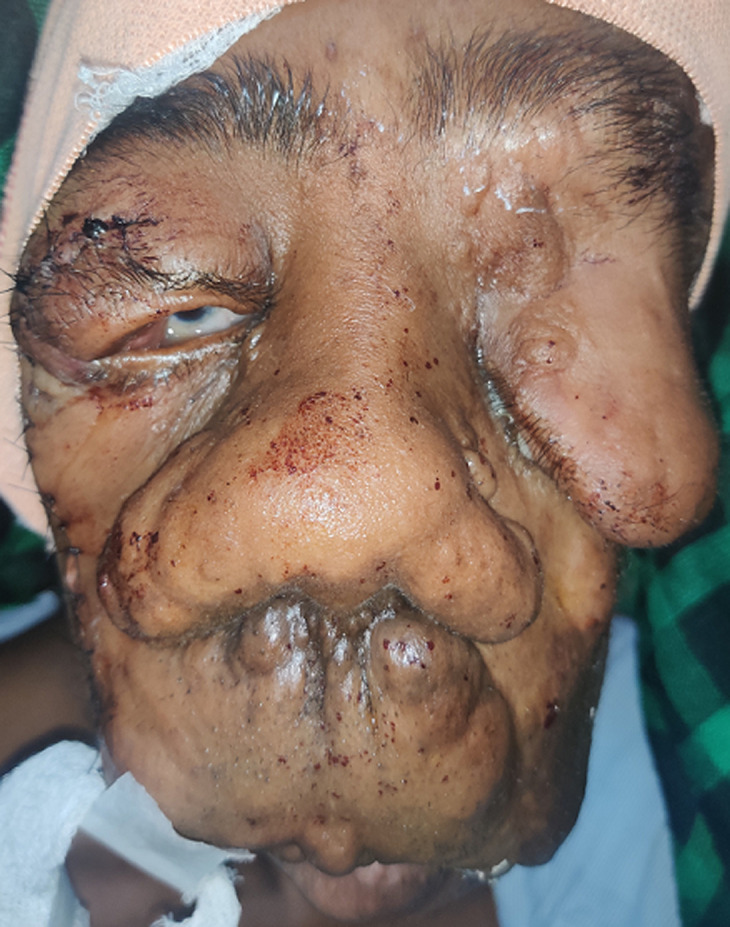
orbito-facial plexiform neurofibromatosis

